# Comparative effectiveness and safety of homologous two-dose ChAdOx1 versus heterologous vaccination with ChAdOx1 and BNT162b2

**DOI:** 10.1038/s41467-022-29301-9

**Published:** 2022-03-23

**Authors:** Eduardo Hermosilla, Ermengol Coma, Junqing Xie, Shuo Feng, Carmen Cabezas, Leonardo Méndez-Boo, Francesc Fina, Elisabet Ballo, Montserrat Martínez, Manuel Medina-Peralta, Josep Maria Argimon, Daniel Prieto-Alhambra

**Affiliations:** 1 Direcció assistencial d’Atenció Primària i a la Comunitat, Institut Català de la Salut (ICS), Generalitat de Catalunya, Barcelona, Spain; 2 Idiap Jordi Gol, Universitat Autonoma de Barcelona, Barcelona, Spain; 3 Centre for Statistics in Medicine, NDORMS, University of Oxford, Oxford, UK; 4 Oxford Vaccine Group, Department of Paediatrics, University of Oxford, Oxford, UK; 5 Public Health Secretariat, Department of Health, Generalitat de Catalunya, Barcelona, Spain; 6 Department of Medical Informatics, Erasmus University Medical Center, Rotterdam, Netherlands

**Keywords:** Epidemiology, Health policy, Vaccines, SARS-CoV-2

## Abstract

Small trials have suggested that heterologous vaccination with first-dose ChAdOx1 and second-dose BNT162b2 may generate a better immune response than homologous vaccination with two doses of ChAdOx1. In this cohort analysis, we use linked data from Catalonia (Spain), where those aged <60 who received a first dose of ChAdOx1 could choose between ChAdOx1 and BNT162b2 for their second dose. Comparable cohorts were obtained after exact-matching 14,325/17,849 (80.3%) people receiving heterologous vaccination to 14,325/149,386 (9.6%) receiving homologous vaccination by age, sex, region, and date of second dose. Of these, 464 (3.2%) in the heterologous and 694 (4.8%) in the homologous groups developed COVID-19 between 1st June 2021 and 5th December 2021. The resulting hazard ratio (95% confidence interval) is 0.66 [0.59–0.74], favouring heterologous vaccination. The two groups had similar testing rates and safety outcomes. Sensitivity and negative control outcome analyses confirm these findings. In conclusion, we demonstrate that a heterologous vaccination schedule with ChAdOx1 followed by BNT162b2 was more efficacious than and similarly safe to homologous vaccination with two doses of ChAdOx1. Most of the infections in our study occurred when Delta was the predominant SARS-CoV-2 variant in Spain. These data agree with previous phase 2 randomised trials.

## Abstract (web summary)

Different homologous and heterologous vaccination regimens have been used for COVID-19. Here the authors show in a cohort analysis from Catalonia that heterologous vaccination with ChAdOx1 followed by BNT162b2 has better vaccine effectiveness than two doses of ChAdOx1.

## Introduction

The rapid development of vaccines to prevent Coronavirus Disease 2019 (COVID-19) has allowed remarkable progress in the global fight against the SARS-CoV-2 pandemic. As of 27 October 2021, around half of the world’s population had received at least one dose of a COVID-19 vaccine. While 15 vaccines have been approved for use by at least one authority, most countries have received more than four approved vaccines^1^.

The Chimpanzee Adenovirus-vectored Oxford (ChAdOx1) and BioNTech/Pfizer mRNA (BNT162b2) vaccines were among the first approved by the European Medicines Agency for emergency use in the European Union. Both vaccines were tested in large phase 3 randomised controlled trials and found to be highly effective against symptomatic SARS-CoV-2 infection when given as two doses^2–5^. Follow-up studies have demonstrated their clinical effectiveness against severe disease, including preventing hospitalisations and mortality, overall^6,7^ and in previously under-researched populations^8^.

Spanish guidelines initially recommended the use of ChAdOx1 for people aged younger than 60 years due to the under-representation of elderly people in the initial pivotal trials^2^. Key workers were targeted in this initial stage to maximise the impact of vaccination on community transmission^9^. Despite their efficacy, reports of thrombotic events after the first dose of adenovirus-based COVID-19 vaccines led to recommendations for heterologous vaccination for those vaccinated with a first dose of ChAdOx1, i.e. many European authorities recommended the use of BNT162b2 for second doses to avoid further exposure to ChAdOx1. A small randomised controlled trial was rapidly conducted that demonstrated better immunogenicity from heterologous vaccination in Spain^10^, but larger studies on clinical effectiveness and safety are urgently needed^11^.

The Spanish authorities allowed citizens previously vaccinated with a first dose of ChAdOx1 to choose between ChAdOx1 and BNT162b2 for their second dose. The majority chose homologous vaccination with two doses of ChAdOx1^12^. In the absence of phase 3/4 randomised controlled trials, this created a natural experiment for studying the comparative safety and effectiveness of these two vaccination schedules. We leveraged routinely collected health data, including electronic medical records linked to vaccination data and laboratory tests, to study the comparative effectiveness and safety of homologous (two-dose ChAdOx1) and heterologous (ChadOx1 followed by BNT162b2) vaccination.

## Results

**Fig. 1:**
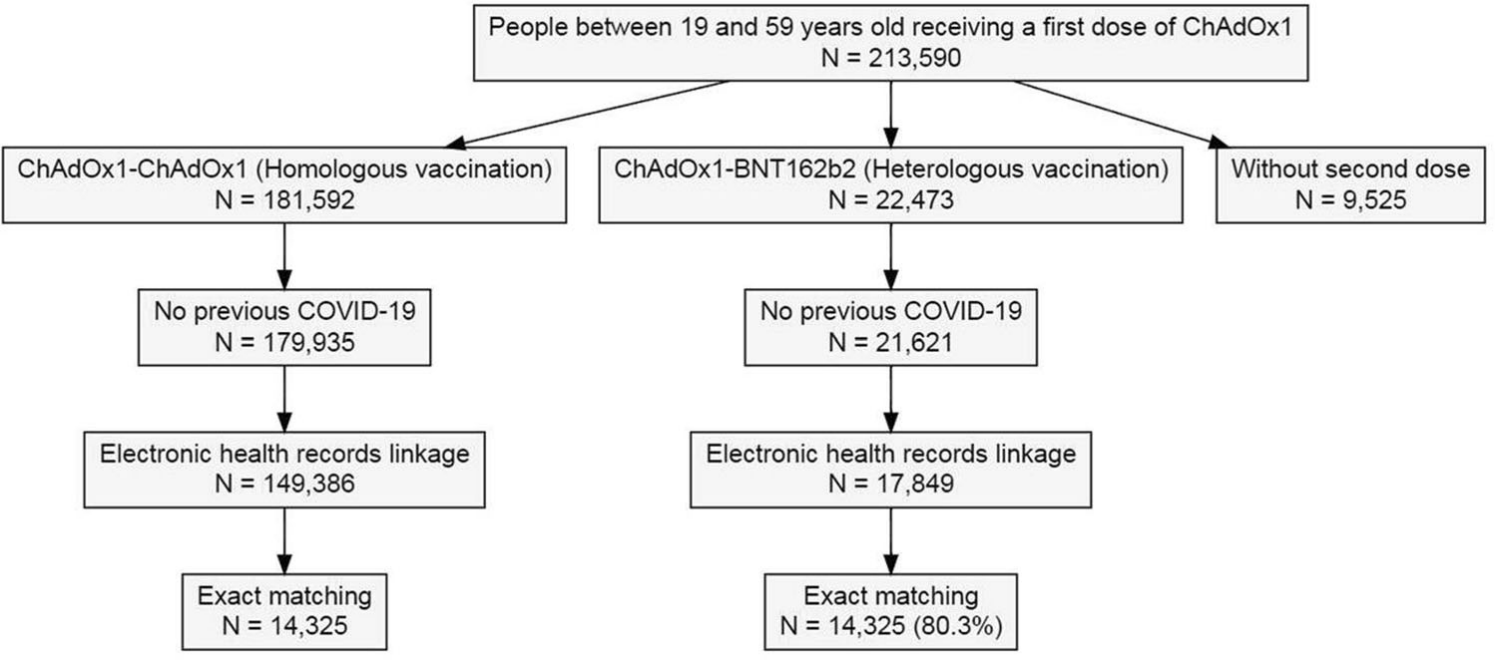
Population flowchart.

Of 167,235 eligible people, 17,849 (10.7%) chose heterologous vaccination and 149,386 (89.3%) chose homologous vaccination. Figure 1 shows the inclusion/exclusion steps used to identify the study participants. For primary analyses, 14,325/17,849 (80.3%) people in the heterologous group were matched to 14,325/149,386 (9.6%) in the homologous group vaccinated with their second dose on the same date (±2 days). The resulting cohorts were comparable in terms of all observed demographics, comorbidity, medicine use, area of residence and socio-economic status (Supplementary Fig. 1). Exact matching ensured the same average (SD) age (42.2 (9.6) years) and proportion of female participants (62.5%) in the two groups. A comparable proportion lived in the most socio-economically deprived (15.8% heterologous vs 15.9% homologous) and rural (19.4% heterologous vs 19.5% homologous) areas of the country (Table 1).

**Table 1:** Baseline characteristics of study participants according to vaccination schedule.

Variable	Heterologous	Homologous
N	14,325	14,325
**Socio-demographic and socio-economic**		
Mean (SD) age, years	42.20 (9.60)	42.21 (9.57)
Female sex	8959 (62.5%)	8959 (62.5%)
Socio-economic status: first quartile (least deprived)	2725 (19.02%)	2745 (19.16%)
Socio-economic status: second quartile	4403 (30.74%)	4363 (30.46%)
Socio-economic status: third quartile	2162 (15.09%)	2145 (14.97%)
Socio-economic status: fourth quartile (most deprived)	2260 (15.78%)	2276 (15.89%)
Residence in a rural area	2775 (19.37%)	2796 (19.52%)
**Medicines use**		
Analgesics	654 (4.57%)	533 (3.72%)
Sedatives/hypnotics	1049 (7.32%)	948 (6.62%)
Anticoagulants	201 (1.40%)	116 (0.81%)
Antidepressants	1228 (8.57%)	1077 (7.52%)
Antiepileptics	449 (3.13%)	353 (2.46%)
Antipsychotics	281 (1.96%)	157 (1.10%)
Antacids	618 (4.31%)	533 (3.72%)
Systemic corticosteroids	101 (0.71%)	82 (0.57%)
Oral antidiabetics	187 (1.31%)	150 (1.05%)
Insulin	105 (0.73%)	73 (0.51%)
Lipid modifying agents	453 (3.16%)	410 (2.86%)
Alpha blockers	5 (0.03%)	2 (0.01%)
Other antihypertensives	4 (0.03%)	1 (0.01%)
Beta blockers	202 (1.41%)	193 (1.35%)
Calcium channel blockers	135 (0.94%)	101 (0.71%)
Combination antihypertensives	209 (1.46%)	176 (1.23%)
Diuretics	103 (0.72%)	108 (0.75%)
ACE inhibitors/ARBs	423 (2.95%)	424 (2.96%)
Chronic obstructive pulmonary disease/asthma inhalers	579 (4.04%)	528 (3.69%)
**Comorbidities**		
Atrial fibrillation	23 (0.16%)	19 (0.13%)
Osteoarthritis	513 (3.58%)	533 (3.72%)
Asthma	935 (6.53%)	910 (6.35%)
Ischaemic heart disease	48 (0.34%)	38 (0.27%)
Diabetes mellitus	266 (1.86%)	209 (1.46%)
Liver disease	289 (2.02%)	278 (1.94%)
Hypertension	826 (5.77%)	814 (5.68%)
Heart failure	5 (0.03%)	2 (0.01%)
Cerebrovascular disease	41 (0.29%)	26 (0.18%)
Chronic obstructive pulmonary disease	46 (0.32%)	37 (0.26%)
Chronic kidney disease	44 (0.31%)	54 (0.38%)
Cancer (all except non-melanoma skin cancer)	344 (2.40%)	325 (2.27%)
Obesity	1539 (10.74%)	1391 (9.71%)
Valvular disease	73 (0.51%)	64 (0.45%)
Hepatitis B	20 (0.14%)	15 (0.10%)
Hepatitis C	49 (0.34%)	34 (0.24%)
HIV infection	49 (0.34%)	47 (0.33%)

**Fig. 2:**
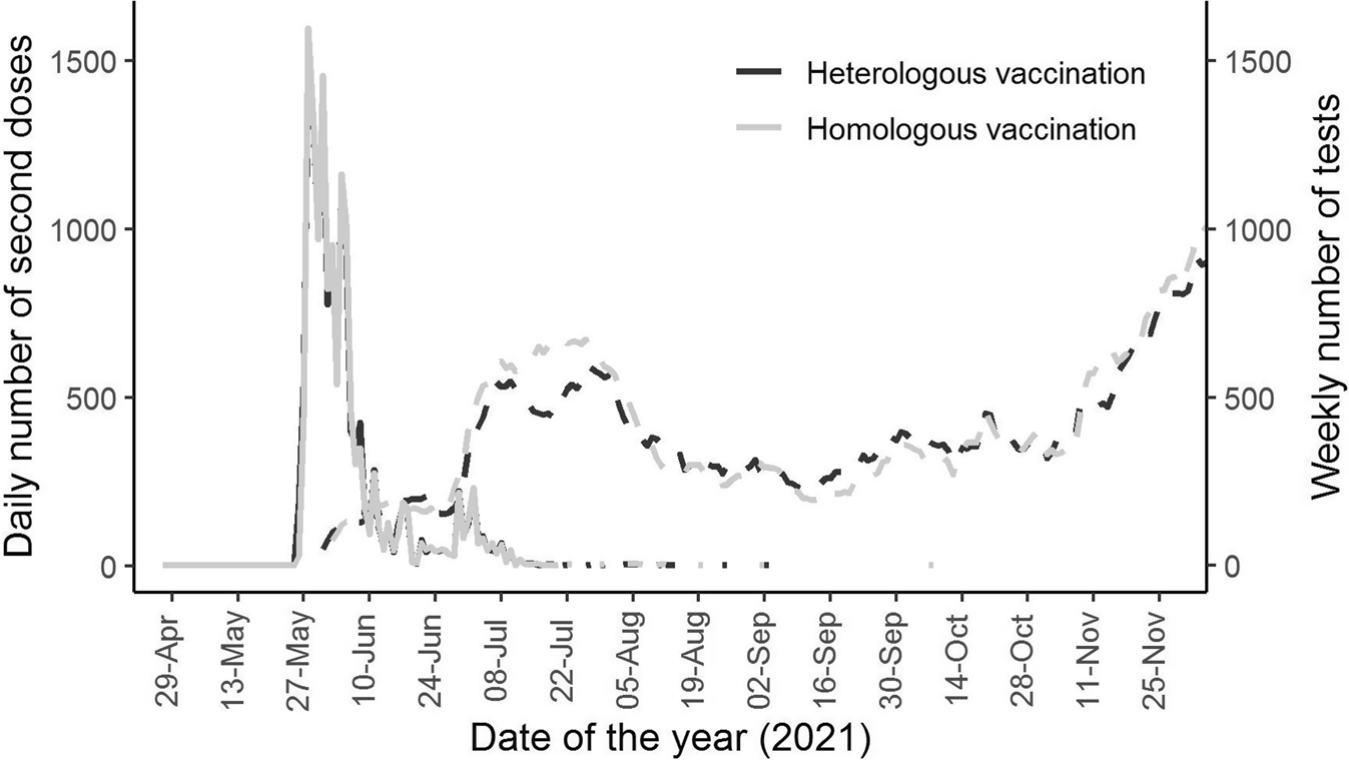
Vaccine uptake and testing rates according to vaccination schedule.

Study participants received their second doses between 27 April 2021 and 8 October 2021. Second doses occurred on the same date for 8742 (61.0%) matched pairs, 1 day apart (before/after) for 4302 (30.0%) pairs and 2 days apart for the remaining 1281 (9.0%) pairs. Test rates were similar in the matched cohorts, with 4874 (34.0%) people on the heterologous schedule and 3268 (35.8%) on the homologous schedule tested at least once during the study period. The average (SD) number of tests for heterologous and homologous groups was 0.82 (1.65) vs 0.87 (1.68) overall and 2.42 (2.04) vs 2.44 (2.03) among those tested at least once during follow-up, respectively. Table 2 shows number, type of test (lateral flow test (LFT) vs polymerase chain reaction (PCR) test) and test incidence rates according to vaccination regimen. The incidence rate ratio [95% confidence interval] of testing for heterologous versus homologous vaccination was 1.01 [0.96–1.06]. Figure 2 shows that the matched cohorts had similar timings for vaccination and testing over time.

**Table 2:** Number and incidence rate (per 1000 person-years) of tests and SARS-CoV-2 infection (positive test) following second-dose vaccination according to vaccination schedule.

	Heterologous		Homologous		HR/IRR
	N / Mean	IR / SD	N / Mean	IR / SD	[95% CI]
Tested	4874	2.21/1000 py	5134	2.35/1000 py	n/a
Number of tests (overall)	0.82	1.65	0.87	1.68	1.01 [0.96–1.06]
N of tests among the tested	2.42	2.04	2.44	2.03	n/a
N of PCR tests (overall)	0.37	1.15	0.38	1.20	1.02 [0.96–1.08]
N of PCR tests among the tested	1.86	1.98	1.87	2.08	n/a
N of LFT tests overall	0.45	1.03	0.50	1.05	1.00 [0.96–1.04]
N of LFT tests among the tested	2.18	1.17	2.17	1.07	n/a
SARS-CoV-2 infection	464	0.18/1000 py	694	0.27/1000 py	0.66 [0.59–0.74]

*CI* confidence interval, *HR* hazard ratio, *IR* incidence rate, *IRR* incidence rate ratio, *N* number, *py* person-years of follow-up, *SD* standard deviation.

**Fig. 3:**
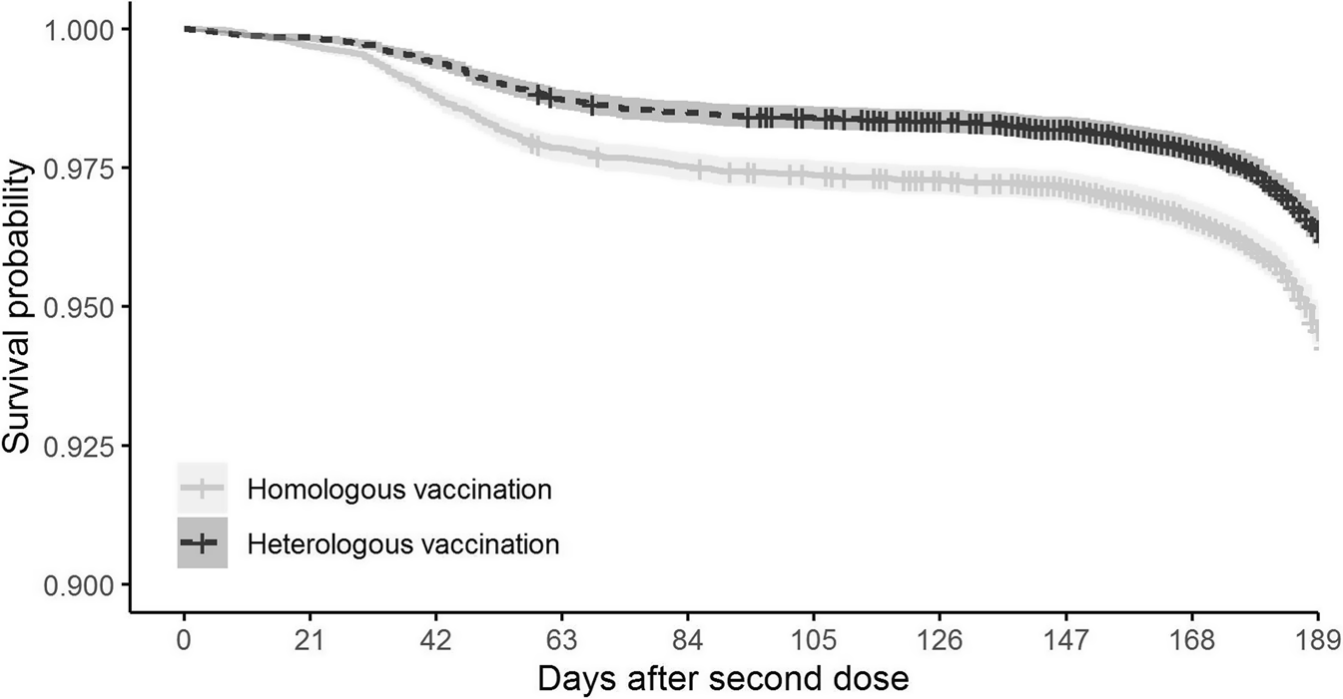
Kaplan–Meier plot of COVID-19 infection (primary outcome) after second-dose vaccination according to vaccination schedule.

Between 1 June and 5 December 2021, SARS-CoV-2 infections were recorded for 464 (3.2%) people in the heterologous group, equivalent to an incidence rate of 0.18/1000 person-years, and 694 (4.8%) people in the homologous group, equivalent to an incidence rate of 0.27/1000 person-years. These rates are equivalent to a hazard ratio of 0.66 [0.59–0.74], favouring heterologous vaccination (Fig. 3 and Table 2). This was equivalent to an absolute risk reduction (ARR) of 0.016 [0.012–0.021] in the study period. Only 2 (0.01%) hospital admissions with COVID-19 were identified in the heterologous group, compared with 5 (0.03%) in the homologous. No deaths were seen in either group. A total of 489 (3.4%) and 318 (2.2%) of the participants in the heterologous and homologous cohorts received a third dose of an mRNA vaccine during follow-up, respectively, and were censored on that date.

Regarding safety, primary analyses of 14,325 people per group found only one venous thromboembolism event (0.007%) and one venous thromboembolism with thrombocytopenia event (0.007%), both in the heterologous group. No myopericarditis events were seen in either group (Table 3).

**Table 3:** Number (%) of safety events in the 21 days following second dose, according to vaccination schedule.

	Heterologous			Homologous		
	1:1 matching	1:2 matching	1:5 matching	1:1 matching	1:2 matching	1:5 matching
N participants	14,325	12,512	8569	14,325	25,024	42,845
N (%) of venous thromboembolism events	1	1	1	0	0	0
N (%) of venous thromboembolism with thrombocytopenia	1	1	1	0	0	0
N (%) of myopericarditis	0	0	0	0	0	1

Back pain episodes were used as a negative control outcome. They were recorded at similar frequencies in the two groups (Supplementary Fig. 2).

Sensitivity analyses were conducted using 1:2 and 1:5 exact matching, resulting in 12,512 people with heterologous vaccination matched to 25,024 people with homologous vaccination and 8569 matched to 42,845, respectively. The hazard ratios for SARS-CoV-2 infection were comparable to those in the primary analysis: 0.65 [0.58–0.73] in the 1:2 and 0.65 [0.57–0.73] in the 1:5 matched cohorts. No safety concerns were identified in these larger cohorts, with only one additional safety event of myopericarditis identified in the homologous vaccination group for 1:5 matching (Table 3).

Finally, a post-hoc analysis was conducted using propensity scores and vaccine date matching instead of the matching described above (see Methods). Comparable cohorts were obtained after matching 17,173/17,849 (96.0%) of those vaccinated with heterologous regimen to 17,173/149,386 (11.5%) in the homologous (Supplementary Fig. 3). A total of 551/17,173 (3.2%) and 809/17,173 (4.7%) COVID-19 cases were identified amongst those in the heterologous and homologous vaccination cohorts, respectively, equivalent to incidence rates of 0.18/1000 and 0.27/1000 person-years. The resulting HR was similar to that seen in the main analysis: HR 0.67 [0.60–0.75].

## Discussion

To the best of our knowledge, this is the first report to date comparing the safety and effectiveness of homologous vaccination against COVID-19 with two-dose ChAdOx1 and heterologous vaccination with first-dose ChAdOx1 and second-dose BNT162b2. Our primary analysis included over 28,000 people, over 14,000 per group, exactly matched on age, sex, region and date of second-dose vaccination. In this rich linked cohort, we found a 34% relative risk reduction of SARS-CoV-2 infection (primary outcome) among those on the heterologous vaccination schedule compared with those on the homologous vaccination schedule, despite similar testing rates in the two groups.

No safety concerns were identified, with only one event (<0.01%) of venous thromboembolism and one event of venous thromboembolism with thrombocytopenia in the heterologous group in the main analysis. One additional event of myopericarditis was observed in 42,845 people receiving homologous vaccination in the 1:5 matched sensitivity analysis.

Sensitivity analyses using 1:2 and 1:5 matching increased the sample size to >37,000 and >50,000 participants, respectively, and confirmed the safety and effectiveness findings. Similarly, a post-hoc analysis using propensity score matching methods yielded consistent findings.

The null association between vaccination schedule and our chosen negative control outcome of back pain supported the robustness of our findings, ruling out residual confounding.

Our findings that heterologous vaccination was more effective than homologous vaccination against COVID-19 agrees with emerging efficacy evidence based on immunological endpoints. Two small, randomised trials have reported higher immunogenicity, characterised by humoral and cellular responses, from ChAdOx1/BNT162b2 than ChAdOx1/ChAdOx1^10,13^. These results were also corroborated by several cohort studies^14–16^. Although people vaccinated with heterologous ChAdOx1/mRNA vaccine (e.g. BNT162b2) were reported to have a 68% lower risk of symptomatic COVID-19 infection than unvaccinated people^17^, little was known about the comparative effectiveness of the heterologous and homologous vaccination schedules against clinical endpoints. Our study showed that heterologous ChAdOx1/BNT162b2 vaccination conferred 34% more protection against SARS-CoV-2 infection than homologous two-dose ChAdOx1 vaccination, and corroborate the potential of immunological surrogate endpoints of COVID-19 vaccines being predictive for clinical protection^18,19^.

Data on the post-marketing safety of heterologous vaccination schedules remain sparse, particularly for rare safety events, with most evidence from evaluations of reactogenicity^10,13–15,20^. Although reactogenicity endpoints are informative for assessing potential vaccine side effects, these trials are underpowered to study rare safety outcomes. Adverse events related to the ChAdOx1 vaccine include the rare (<1/1000 to ≥1/10,000) outcome venous thrombosis and the very rare (<1/10,000) outcome vaccine-induced immune thrombosis with thrombocytopenia syndrome^21,22^. Similarly, myocarditis and pericarditis outcomes possibly associated with BNT162b2 are expected to affect around 10–24 people per 10 million fully vaccinated people aged ≥30 years^23^. In this study with >28,000 participants, we identified one venous thromboembolism event and one venous thromboembolism with thrombocytopenia event. The number of events did not increase much in sensitivity analyses including up to >50,000 participants, suggesting that larger studies are needed to investigate these safety signals for heterologous vaccination schedules.

Our study has several limitations. The main limitation is the observational nature of our data. However, exact matching on age, region, and date led to a good balance in all observed confounders, including socio-demographics, comorbidity and medicines use. Propensity score matching was used in a post-hoc analysis to confirm the robustness of our exact-matching approach. Additionally, our analysis of a negative control outcome (back pain) suggested comparability of the matched cohorts, including unobserved covariates.

As most of our participants were middle-aged adults aged less than 60 years old, our risk-benefit assessment may not be valid for younger or elderly people. In addition, we could not include the cohort of people receiving 2 doses of BNT162b2 in our analyses, as they were vaccinated during a different calendar period, as depicted in Supplementary Fig. 4. Failing to match on vaccination date would lead to biased results due to changes in community transmission during the study period. Finally, our sample size was insufficient for studying severe COVID-19 outcomes, including hospitalisation and mortality, or rare safety outcomes.

This study also has strengths. The rich, representative linked dataset used allowed a robust analysis of vaccine exposure and outcomes at speed to inform ongoing international vaccination campaigns. Catalonia has a universal healthcare system and uses a centralised, secure data ecosystem with a long track record of research and high-impact publications^8,24^. The granularity of these data made it possible to control for confounding and test for residual systematic bias. Linkage to additional data sources on RT-PCR and LFT tests allowed for a comprehensive assessment of testing rates and reliable SARS-CoV-2 infection rates. The data collection period covered a time when most cases of COVID-19 in Catalonia were attributable to the Delta variant of SARS-CoV-2^25^,which is still the predominant variant worldwide. Our data are highly relevant for ongoing global vaccination strategies and future and current third-dose and booster campaigns.

In conclusion, by leveraging the potential of multiple data sources in parallel, our study confirmed that a heterologous vaccination schedule of ChAdOx1 and BNT162b2 was safe and provided better protection against COVID-19 than a homologous ChAdOx1 vaccination schedule in real-world settings experiencing the Delta variant. More research on other mixed-vaccine schedules with different prime-boost intervals are needed.

## Methods

### Study design and data sources

We performed a cohort study based on linked routinely collected data available to the Public Health Secretariat of Catalonia. Vaccine exposure was obtained from the Catalan Shared Clinical Records, a database with vaccine data covering the entire Catalan health system and all its vaccination centres. Additional linked data were obtained from the Catalan database of reverse transcription polymerase chain reaction (RT-PCR) tests and lateral flow tests (LFT) for SARS-CoV-2, from primary-care electronic health records and from a population-based administrative hospital admissions data (CMBD-AH for its acronym in Catalan language). Data from these linked databases have previously been used for multiple COVID-19 research studies and include information for nearly 90% of the Catalan population^8^.

### Participants, cohorts and follow-up

For our primary analysis, we included all individuals aged 19–59 years old who received a first dose of the ChAdOx1 vaccine and a second dose of ChAdOx1 (homologous vaccination) or BNT162b2 (heterologous vaccination). We followed participants from the day they received their second dose of either vaccine until an outcome, death, third dose of the vaccine, or the end of data availability (5 December 2021).

We excluded people with a previous SARS-CoV-2 infection identified by a positive RT-PCR test or LFT and people assigned to one of the 10% of primary-care practices not contributing to our database.

Each participant receiving heterologous vaccination was matched 1:1 to one person receiving homologous vaccination using exact matching by age, sex, general practice centre and date of second dose. In a sensitivity analysis, we changed the matching ratio to 1:2 and 1:5 to increase sample size.

### Study outcomes

The primary outcome for effectiveness analyses was SARS-CoV-2 infection, defined by the date of the earliest of a positive RT-PCR test or LFT, regardless of symptoms or clinical diagnosis. We measured the number of tests over time regardless of results as an additional outcome to account for diagnostic effort.

Safety outcomes included venous thromboembolism, venous thromboembolism with thrombocytopenia and myopericarditis within 21 days after the second vaccine dose, based on ChAdOx1^21^ and BNT162b2^23^ safety reports. Supplementary Table S2 includes the ICD-10-CM codes (international classification of diseases, 10th revision, clinical modification) used to ascertain when these events occurred.

We analysed the occurrence of a negative control outcome—low back pain—to identify potential unmeasured confounding. Negative control outcomes are health events not causally associated with the exposure of interest, here vaccination.

### Additional covariates

Covariates used for confounding assessment included socio-demographics and clinical features assessed at the time of inclusion (day of the second vaccination), as recorded in primary-care electronic health records and linked administrative data: age (in years), sex, area of residence, rurality and socio-economic status, number of RT-PCR tests or LFT performed, pre-existing comorbidities and long-term medicine use. Supplementary Table 1 provides ICD-10-CM codes for comorbidities and Anatomical Therapeutic Chemical Classification (ATCC) codes used to identify previous medicine use. We assessed socio-economic status using a validated deprivation index based on census data (MEDEA deprivation index)^26,27^. Rurality of residence was measured, with rural areas defined by a population <10,000 inhabitants and a density <150 inhabitants/km^2^, as per regional guidance.

### Statistical analysis

Exact matching (1:1) between heterologous ChAdOx1/BNT162b2 vaccination and homologous two-dose ChAdOx1 was performed using the following variables: age (±2 years), sex, general practice centre and day of the second vaccination (±2 days). As a sensitivity analysis, we generated additional study populations and repeated all analyses after matching with 1:2 and 1:5 ratios. We assessed confounding due to known variables by measuring covariate imbalance as the standardised mean difference (SMD) of all covariates listed above. We considered SMD > 0.1 to be imbalanced^28^.

Separately, we used propensity score matching as part of a post-hoc analysis requested by one of the manuscript reviewers. Propensity scores were estimated using logistic regression including all available confounders, and cohorts matched using propensity score values with a maximum caliper width of 0.2 SD, with additional exact matching on second-dose vaccination date (±2 days).

We plotted time-to-event Kaplan–Meier estimates according to vaccine exposure (homologous vs heterologous). Absolute risk reduction (ARR) was estimated as the difference in cumulative incidence of Covid-19 amongst those receiving homologous–heterologous vaccination. Cox regression models were then fitted to calculate hazard ratios and 95% confidence intervals for each of the study outcomes, according to vaccination schedule. Visual inspection of Schoenfeld residuals against the transformed time was used to evaluate the proportionality of hazards. A zero-inflated negative binomial regression model was used to calculate the incident rate ratio and 95% confidence interval for the number of tests.

All analyses were conducted using R version 4.0.0.

#### Ethical considerations and information governance

All data were obtained from linked administrative sources after pseudonymisation in accordance with articles 6. e), 9.2. j) + 89 RGPD and 17.2.d of the LOPD-GDD. The proposed study was evaluated and approved by the Clinical Research Ethics Committee of the IDIAP Jordi Gol with Reference 21/269-PCV. This research was based on the agreement established in Regulation 2016/679 of the European Parliament and of the Council of April 27, 2016 on Data Protection and Organic Law 3/2018 of December 5 of protection of personal data and guarantee of digital rights.

### Reporting summary

Further information on research design is available in the Nature Research Reporting Summary linked to this article.

## Supplementary information

Supplementary Information

Peer Review File

Description of Additional Supplementary Files

Supplementary Data 1

Supplementary Data 2

Supplementary Data 3

Supplementary Code 1

Reporting Summary

## Data Availability

Patient-level data utilised for the reported analyses are not accessible openly due to local information governance regulations as defined in the ‘Ethical considerations and information governance’ section. Aggregated data are provided for inspection as Supplementary Data 1-3, and R code used for analyses as Supplementary Code 1.
