# Supplementary material for: Comparative effectiveness and safety of homologous two-dose ChAdOx1 versus heterologous vaccination with ChAdOx1 and BNT162b2
Source: Nat Commun. 2022 Mar 23;13:1639. doi: 10.1038/s41467-022-29301-9 (PMC8943099; doi:10.1038/s41467-022-29301-9)

SUPPLEMENTARY FIGURES AND TABLES

Supplementary Table 1. Co-morbidities and long-term medications included in the analysis.

**Comorbidity**	**ICD10-CM codes**
Asthma	J45, J45.20, J45.21, J45.22
Cancer (all except non-melanoma skin cancer)	C00-C97 (except C44), D00-D09
Atrial fibrillation	I48.0. I48.1, I48.2, I48.91
Cerebrovascular disease	G45.0, G45.1, G45.2, G45.8, G45.9, G46.0, G46.1, G46.2, G46.3, G46.4, G46.5, G46.6, G46.7, G46.9, I63.00, I63.10, I63.20, I63.30, I63.40, I63.50, I63.6, I63.8, I63.9, I67.82, I67.9, I69.30, I69.320 I69.398, I69.80, I69.998<br>I61.0, I61.1, I61.2, I61.3, I61.4, I61.5, I61.6, I61.8, I61.9, I61.10
Chronic Kidney Disease	E66.01, E66.09, E66.1, E66.2, E66.8, E66.9
Chronic obstructive pulmonary disease	J43.0, J43.1, J43.2, J43.8, J43.9, J44, J44.0, J44.1, J44.9
Diabetes mellitus (type I and II)	E10.10, E10.29, E10.311, E10.359, E10.39, E10.49, E10.59, E10.621, E10.65, E10.69, E10.8, E10.9<br>E11.01, E11.21, E11.22, E11.29, E11.311, E11.39, E11.40, E11.43, E11.49, E11.51, E11.59, E11.610. E11.621, E11.638, E11.641, E11.649, E11.65, E11.69, E11.8, E11.9, E13.10, E13.29, E13.39, E13.49, E13.59, E13.641, E13.69, E13.8, E13.9
Heart failure	I50.1, I50.20, I50.30, I50.32, I50.9
HIV infection	Z21, B20, B97.35
Hypertension	I10, I11.0, I11.9, I12.0, I12.9, I13.0, I13.10, I13.2, I15.0, I15.1, I15.2, I15.8, I15.9
Ischaemic heart disease	I20, I20.0, I20.8, I20.9, I24.0, I24.1, I24.8, I24.9, I25, I25.10, I25.2, I25.41, 125.5, I25.6, I25.89, I25.9, I70.90, I21, I21.0, I21.01, I21.02,I21.09, I21.1, I21.11, I21.19, I21.2, I21.29, I21.3, I21.4, I22.0, I22.1, I22.2, I22.8, I22.9, I23.0, I23.1, I23.2, I23.3, I23.4, I23.5, I23.6, I23.8
Liver disease	K70.0, K70.10, K70.2, K70.30, K70.9, K73.0, K73.1, K73.2, K73.8, K73.9, K75.0, K75.2, K75.3, K75.4, K75.89, k75.9, K76.5, K76.7, K76.9

Obesity	E66.01, E66.09, E66.1, E66.2, E66.8, E66.9
Type B hepatitis	B16.0, B16.1, B16.2, B16.9
Type C hepatitis	B17.10, B18.2
**Medicine or drug class**	**ATC codes**
Analgesics	M01AX%, N02AA%, N02AB%, N02AC%, N02AD%, N02AE%, N02AX%, N02BA%, N02BB%, N02BE%<br>M01AA%, M01AB%, M01AC%, M01AE%, M01AG%, M01AH%.
Sedatives/hypnotics	N05BA%, N05BB%, N05BE%, N05BX%, N05CD%, N05CF%, N05CM%,
Anticoagulants	B01AA%, B01AB%, B01AC%, B01AE%, B01AF%, B01AX%
Antidepressants	N06AA%, N06AB%, N06AG%, N06AX%
Antiepileptics	N03AA%, N03AB%, N03AC%, N03AD%, N03AE%, N03AF%, N03AG%, N03AX%
Anti-psychotics	N05AA%, N05AB%, N05AC%, N05AD%, N05AE%, N05AF%, N05AH%, N05AX%, N05AK%, N05AL%
Antiacids	A02AB%, A02AD%, A02AX%, A02BA%, A02BB%, A02BC%, A02BX%,
Systemic corticoids	H02AB%, H02BX91
Oral antidiabetics agents	A10BA%, A10BB%, A10BD%, A10BF%, A10BG%, A10BH%, A10BX%, A10BK%, A10BJ%
Insulin	A10AB%, A10AC%, A10AD%, A10AE%
Lipid modifying agents	C10AA%<br>C10AB%, C10AC%, C10AD%, C10AX%, C10BA%, C10BX%,
Alpha blockers	C02CA%
Beta blockers	C07AA%, C07AB%, C07AG%
Calcium channel blockers	C08CA%, C08DA%, C08DB%

Diuretics	C03AA%, C03BA%, C03DA%, C03DB%, C03EA%, C03EB%
ACEI/ARBs	C09AA%, C09CA%
Inhalers	R03AA%, R03AC%, R03AK%, R03AL%, R03BA%, R03BB%

Supplementary Table S2. Diagnostic codes (ICD-10) of adverse events considered in the study

**Adverse event**	**ICD-10 codes**
Venous thromboembolism (VTE), includes cerebral venous sinus thrombosis, mesenteric thrombosis, portal vein thrombosis, non-limb venous thrombosis, venous thrombosis of extremities, non-specific venous thrombosis, pulmonary embolism	G08, I676, I636, K55011, K55012, K55019, K55021, K55022, K55029, K55031, K55032, K55039, K55041, K55042, K55049, K55051, K55052, K55059, K55061, K55062, K55069, I81, I821, I82210, I82220, I82290, I823, I82B11, I82B12, I82B13, I82B19, I82C11, I82C12, I82C13, I82C19, I82401, I82402, I82403, I82409, I82411, I82412, I82413, I82419, I82421, I82422, I82423, I82429, I82431, I82432, I82433, I82439, I82441, I82442, I82443, I82449, I82491, I82492, I82493, I82499, I824Y1, I824Y2, I824Y3, I824Y9, I824Z1, I824Z2, I824Z3, I824Z9, I82601, I82602, I82603, I82609, I82611, I82612, I82613, I82619, I82621, I82622, I82623, I82629, I82811, I82812, I82813, I82819, I82A11, I82A12, I82A13, I82A19, I8000, I8001, I8002, I8003, I8010, I8011, I8012, I8013, I80201, I80202, I80203, I80209, I80211, I80212, I80213, I80219, I80221, I80222, I80223, I80229, I80231, I80232, I80233, I80239, I80291, I80292, I80293, I80299, I803, I82890, I8290, I808, I809, I2601, I2602, I2609, I2690, I2692, I2699
Thrombocytopenia	D693, D6959, D696, D7582
Pericarditis/myocarditis	I30, I40, I010, I012, I090, I092, I241, I300, I301, I308, I309, I310, I311, I32, I400, I401, I408, I409, I41, I514, I970, D8685, J1182, A3952, B2682, A3953, B3323, B3322, J1082, M3212, A381, B5881

Supplementary Figure 1. Standardised mean differences (SMD) in the matched study population, showing confounder imbalance

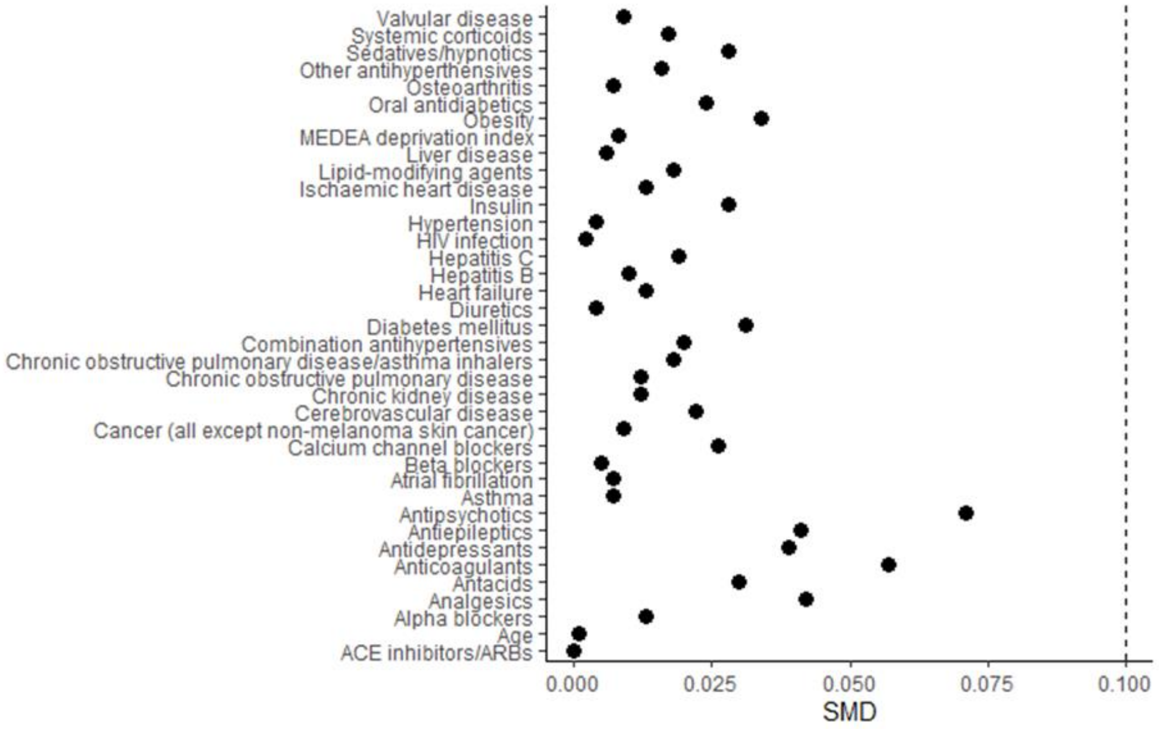

Supplementary Figure 2. Kaplan-Meier of low back pain according to vaccination schedule

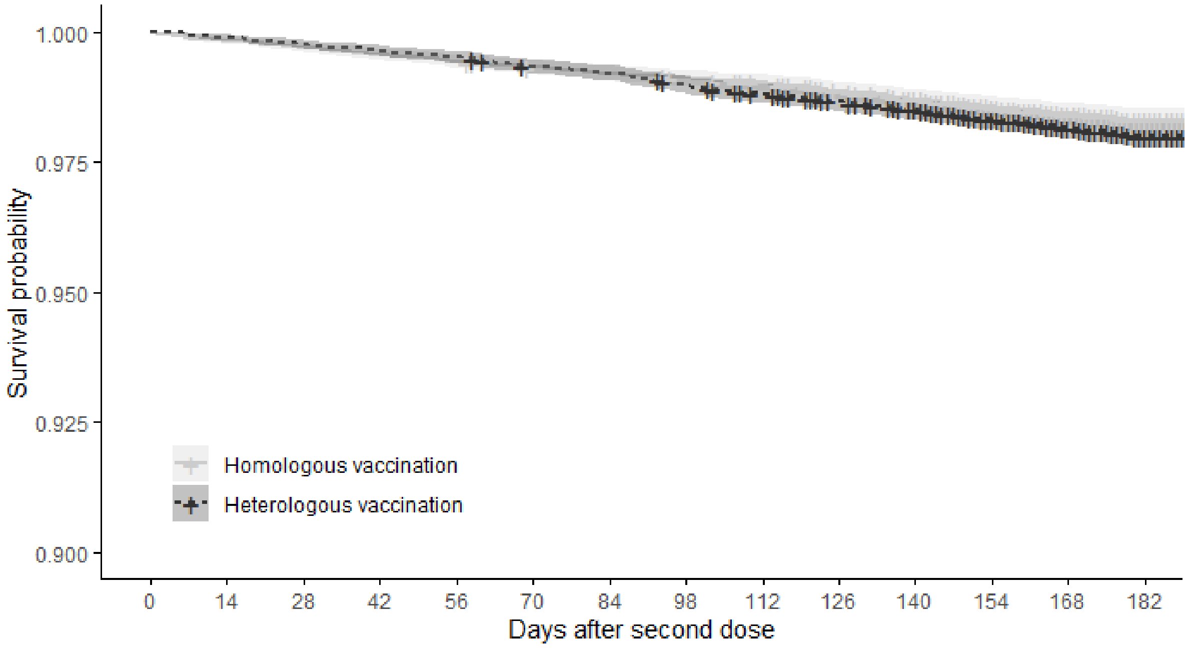

Supplementary Figure 3. Standardised mean differences (SMD) in the propensity score matched cohorts (post-hoc analysis)

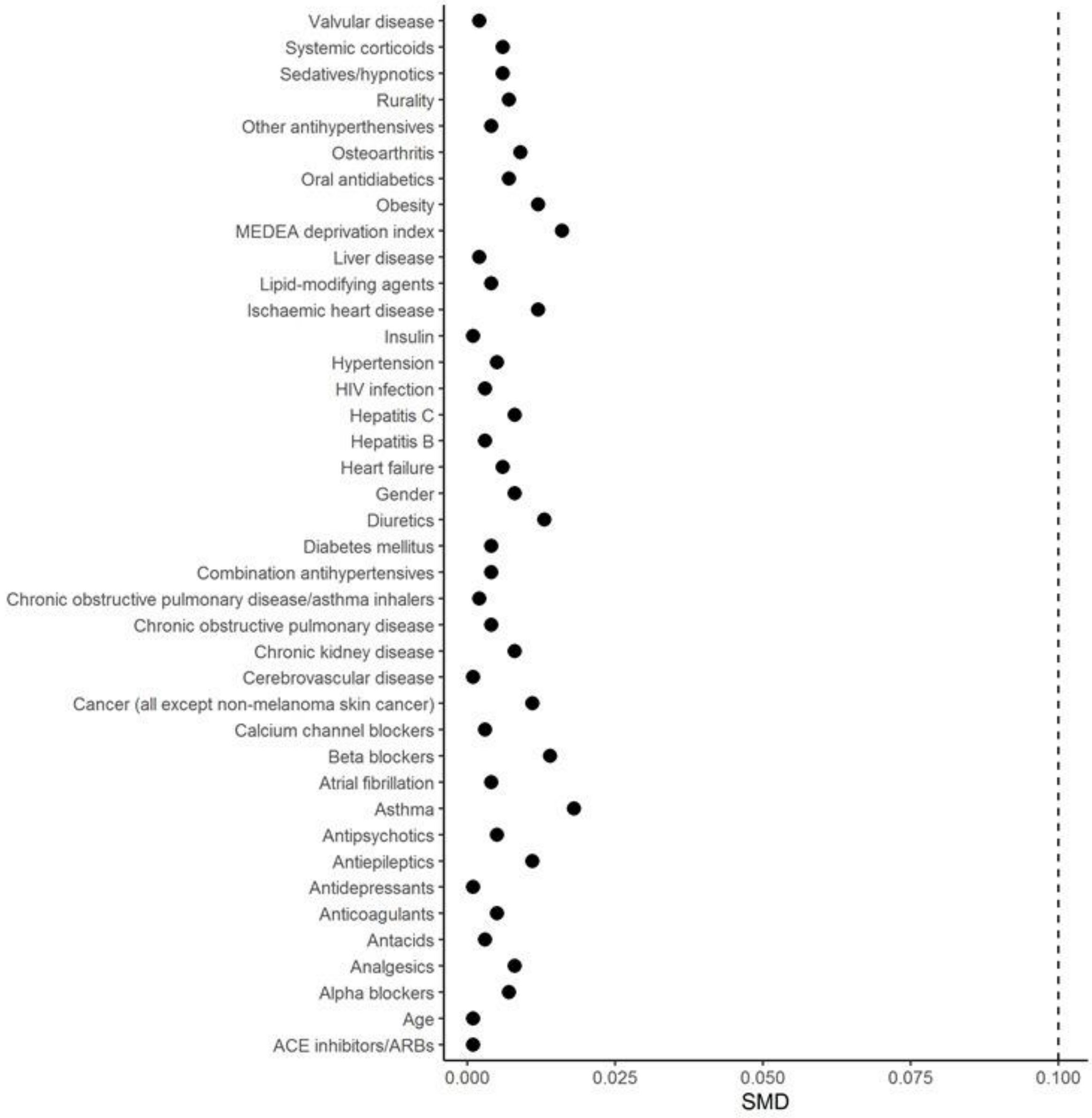

Supplementary Figure 4. Uptake of different vaccination regimens in Catalonia over calendar time during the study period.

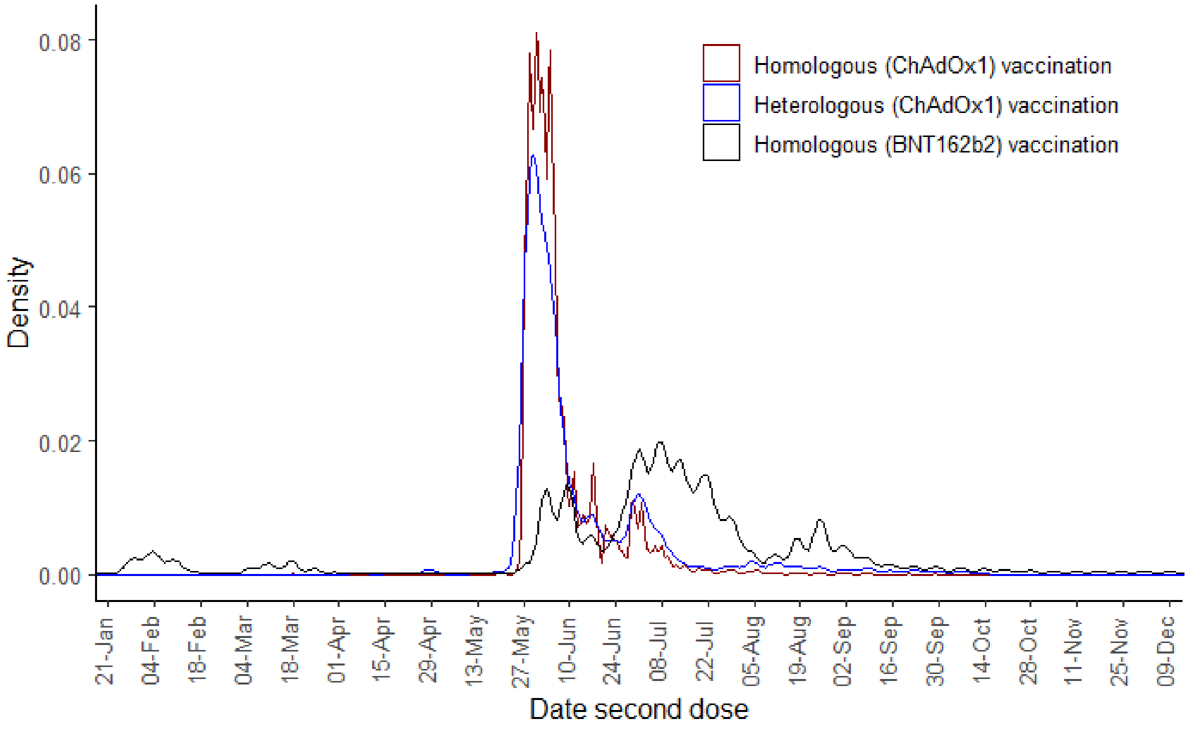

Supplement: Supplementary file 1 — Supplementary Information [file 41467_2022_29301_MOESM1_ESM.pdf]
