# Supplementary material for: Comparative effectiveness and safety of homologous two-dose ChAdOx1 versus heterologous vaccination with ChAdOx1 and BNT162b2
Source: Nat Commun. 2022 Mar 23;13:1639. doi: 10.1038/s41467-022-29301-9 (PMC8943099; doi:10.1038/s41467-022-29301-9)
Supplement: Supplementary file 3 — Description of Additional Supplementary Files [file 41467_2022_29301_MOESM3_ESM.pdf]

File Name: Supplementary Data 1
Description: Aggregated data used to produce Kaplan-Meier estimates, plots, and survival analyses in the primary analysis (matched 1:1)

File Name: Supplementary Data 2
Description: Aggregated data used to produce Kaplan-Meier estimates, plots, and survival analyses in the primary analysis (matched 1:2)

File Name: Supplementary Data 3
Description: Aggregated data used to produce Kaplan-Meier estimates, plots, and survival analyses in the primary analysis (matched 1:5)

File Name: Supplementary Code 1
Description: R code used to execute the study analyses
